# Rising HIV prevalence among men who have sex with men in Nigeria: a trend analysis

**DOI:** 10.1186/s12889-019-7540-4

**Published:** 2019-09-02

**Authors:** George I.E Eluwa, Sylvia B. Adebajo, Titilope Eluwa, Obinna Ogbanufe, Oluwafunke Ilesanmi, Charles Nzelu

**Affiliations:** 1 Population Council, No. 16, Mafemi Crescent, Utako, Abuja, Nigeria; 2 Save the Children International, Abuja, Nigeria; 3 Centers for Disease Prevention and Control, United States Embassy, Abuja, Plot 1038 Diplomatic Drive, Abuja, Nigeria; 4 HIV and Viral Hepatitis Communicable Diseases Cluster, World Health Organization Country Office, Abuja, Nigeria; 5 National AIDS and STI Control Program, Federal Ministry of Health, Abuja, Nigeria

**Keywords:** Men who have sex with men, MSM, HIV, Trend analysis, Nigeria

## Abstract

**Background:**

Men who have sex with men (MSM) are conservatively estimated to be less than 1% of the Nigerian population yet nationally account for about 20% of new HIV infection. We estimated the trend in HIV prevalence and determined correlates of HIV infection among MSM.

**Methods:**

This study used data from respondent-driven sampling in three rounds of integrated biological and behavioral surveillance survey (2007, 2010 and 2014) and covered three states in 2007, six states in 2010 and eight states in 2014. Each round used similar methodology and thus allows for comparison. Behavioral data were obtained using a structured pre-coded questionnaire. Differences in categorical variables were assessed with Chi Square. Logistic regression was used to identify factors associated with HIV.

**Results:**

A total of 879, 1545 and 3611 MSM were recruited in 2007, 2010 and 2014 respectively. Median age was 22 years for 2007 and 2014 while it was 24 years in 2010. About one-third of MSM in 2007 and 2014 and about two-fifths in 2010 had engaged in transactional sex. HIV prevalence increased from 14% in 2007 to 17% in 2010 to 23% in 2014 (*p* < 0.0001). Factors associated with HIV include older age ≥ 25 years (adjusted odds ratio {AOR}:2.41; 95% CI:1.84–3.16); receptive anal sex (AOR:1.92; 95% CI:1.54–2.40) and history of sexually transmitted infections (AOR:1.26; 95% CI:1.02–1.55).

**Conclusion:**

There’s been a consistent and significant increase in HIV prevalence among MSM with about 10-percentage points relative increase per year over 7 years. Older MSM were more likely to be HIV positive and this may reflect their prolonged exposure to high risk sexual activities. Evidence based interventions are urgently needed to mitigate intra-group HIV transmission and propagation of HIV epidemic between MSM and the general population.

## Background

Globally, men who have sex with men (MSM) remain disproportionately infected and affected by HIV [1, 2]. Despite huge investments in global HIV and expanded antiretroviral treatment (ART) programs that have resulted in significant declines in HIV among other sub-populations (general population, female sex workers), HIV among MSM has remained on a sustained increase globally [1, 3]. In high income countries, the trend of HIV epidemic has been on a decline except among MSM [1]. Similarly, available data on HIV incidence and prevalence from low and middle-income countries suggest that the HIV epidemic among gay, bisexual and other men who have sex with men are on a markedly different and increasing trajectory [1–3]. In the USA, new HIV infections among MSM has been estimated to be increasing at 8% per annum since 2001 [1]. In the Amsterdam Cohort Study among MSM, HIV acquisition increased from 1.0 per 100 person-years in 1992 to 2.0 per 100-person years in 2009 [4, 5] while in China, from prospective cohort studies conducted among MSM between 2005 and 2007, HIV acquisition was reported to have increased from 2.6 to 5.6 per 100 person-years [6–9].

Most studies of HIV acquisition and transmission among MSM have largely focused on individual level risk factors including unprotected receptive anal intercourse, high number of lifetime male partners, injecting and non-injecting drug use and high viral load in the index partner [1]. However, individual level risk factors alone, have been shown to be insufficient to explain the high transmission dynamics of HIV among MSM and the divergence of MSM epidemics when compared to HIV epidemics in other populations [1, 2]. Other risk factors such as biological, couple-network level, community-level and structural drivers have been established to be pertinent in understanding the persistent high transmission rates among MSM especially in the presence of increased ART coverage whereby new infections should decrease as a result of reduced likelihood of transmission because of the effect of ART on viral load [2, 10, 11].

MSM in Nigeria are conservatively estimated to be less than 1% of the Nigerian population yet nationally account for about 20% of new HIV infections [12]. MSM are criminalized and stigmatized and this has further worsened in recent years with the passing of the Same-Sex Marriage Prohibition law of 2014 [13–17]. The new law included clauses that prohibited organizations from providing services to MSM and facilitation of meetings that support gay people, thus further criminalizing same-sex activities [12]. Studies have shown that these restrictive policies further limit the poor coverage of HIV prevention, treatment, and care programs among MSM [12, 18–21]. Very few MSM-targeted HIV prevention, treatment and care services exist in a limited number of states in Nigeria. This is chiefly because of limited data on the size estimate of MSM across states as well as limited funding for key population dedicated programs. Data on MSM are largely from three rounds of the population-based Integrated Biological and Behavioural Surveillance Survey (IBBSS) conducted in, 2007, 2010, and 2014. This study aimed to assess the change in HIV prevalence and determine correlates of HIV among MSM in Nigeria. Evidence from this study will be used by policy maker and program managers for evidence-based decision making for HIV prevention among MSM in Nigeria.

## Methods

### Study sites

In 2007, only three states - Lagos, Kano and Cross River, were included in the IBBSS. In 2010, following increased awareness of the contribution of MSM to the HIV epidemic, an additional three states were included in the IBBSS - the Federal Capital Territory (FCT), Kaduna and Oyo, while in 2014, Enugu and Rivers states were added to the six states included in the 2010 IBBSS. The state selection ensured that five of the six geopolitical zones in Nigeria were represented in the survey. Thus, the south west (Lagos and Oyo), south east (Enugu), south south (Rivers), north central (FCT) and north west zones (Kano and Kaduna) contributed to the study.

### Sampling design and recruitment

Each round of the IBBSS used respondent driven sampling given to the hidden nature of MSM and thus allows for comparison between and across rounds. Respondent driven sampling (RDS) has been described in detail in previous studies [22, 23]. Briefly, RDS is a modified chain referral non-random sampling method of recruitment that adjusts for the non-randomness using a mathematical model that weights each sample recruited [23]. Inclusion criteria was male aged 16 years and above with a history of oral and/or anal sexual contact with another man in the 6 months prior to the survey. Known MSM, designated as “seeds” began the chain of the referral network and use vouchers to recruit their peers into the study. Ten seeds were selected for each round and seeds were diversified by age, educational status and socioeconomic status. To avoid an over-representation of MSM with similar attributes, vouchers limited to three per recruit was used [23, 24]. In addition, to avoid repeat enrollment, only one screener was used, only one person was approved to reimburse MSM who had successfully recruited his peers and only one location was used. Each voucher was redeemable and yielded N500 [approx. $3 USD] as an incentive for participating, with an additional N500 given to a recruit for each successful additional participant. Total compensation was limited to a maximum compensation of N2000 [approximately $12 USD]. Sample size for the 2007 IBBSS was estimated based on an assumed HIV prevalence of 15%, a design effect of 2.0 and level of precision of 0.05. For the 2010 and 2014 IBBSS, sample size was estimated to detect a 10% change from the subsequent round.

### Data collection

Structured close-ended interviewer administered questionnaires elicited information on socio-demographic characteristics, type of sex partners and sexual risk behaviors. Interviews were conducted in MSM friendly organizations identified in each of the study states. Transactional sex was assessed both with female and male partners. Type of anal sex practiced was categorized as “insertive penile sex” or “receptive penile sex” in the past 6 months. HIV risk perception was assessed by asking MSM “do you feel you are at risk of infection with HIV?” with response options being “yes or no”. Consistent condom use with sexual partners during transactional and non-transactional sex was assessed by asking the questions “how often did you or your male partner use a condom every time you had sex in the last six months?” while condom use at last sex was assessed by the question “the last time you had anal sex did you or your partner use a condom?”. Transactional sex was assessed by asking “have you received money or gift in exchange for sex in the last 6 months?” Written consent was obtained from all participants for both behavioural and biological components of the survey.

### Laboratory testing

Detection of HIV during all the studies was consistently done by rapid test using whole blood samples obtained from a finger prick. Based on the national HIV testing guideline, a parallel algorithm of Determine (Alere Medical, USA) and Unigold (Trinity Biotech, Plc, Bray, Ireland) was used to identify HIV sero-positivity while status of discordant tests was confirmed with the use of Stat Pak (Chembio Diagnostic Systems, New York, USA).

### Data management and analysis

Data from each study state were entered centrally using CS Pro version 3.2 and 25% of questionnaires underwent double-data entry to ensure data quality. Behavioural and biological data were linked by study unique identification number for each participant. Data from each round were merged and analyzed using STATA 14.0 (STATA Corporation). Descriptive statistics of demographic, behavioural, and biological variables was conducted. The Cochran-Armitage Chi Square trend test was used to compare differences between categorical variables across different rounds of IBBSS, while the Kruskal-Wallis test was used to compare differences in continuous variables across different rounds of IBBSS. To measure associations between HIV and predictor variables (Tables), bivariate logistic regression analysis was conducted with *p* value ≤0∙2 designated as the cutoff for inclusion in multivariate logistic regression models. Variables were retained if they attained a p value ≤0∙05 in the multivariate analysis. The predictor variables were based on data from literature that showed an association between the variables and HIV.

## Results

### Sociodemographics

Table 1 highlights the sociodemographic data of MSM across the three rounds of the IBBSS. A total of 897, 1545 and 3611 MSM were surveyed in 2007, 2010 and 2014 respectively. Many of the respondents were aged 20–24 years in 2007 (61%) and 2014 (46%), while in 2010, almost half (47%) of the respondents were 25 years or older. Over 85% of the respondents had never married in each round of the survey. Majority of the respondents had completed at least secondary level education, and this was highest among respondents in 2007 with 63% followed by respondents in 2014 (55%).

**Table 1:** Sociodemographics, HIV prevalence and sexual risk behaviors among MSM 2007–2010

Characteristics	2007 (*n* = 897)	95% CI	2010 (*n* = 1545)	95% CI	2014 (*n* = 3611)	95% CI	*p* value
	% (n)		% (n)		% (n)		
HIV prevalence	13.5 (109)	11.3–16.0	17.2 (222)	15.2–19.4	22.9 (711)	21.5–24.4	< 0.0001
Age (yrs)							
16–19	13.2 (116)	11.1–15.6	13.8 (213)	12.2–15.6	20.9 (756)	19.6–22.2	
20–24	60.5 (532)	57.2–63.7	39.6 (612)	37.2–42.1	45.9 (1658)	44.3–47.5	
≥ 25	26.3 (231)	23.5–29.3	46.6 (720)	41.1–49.1	33.2 (1197)	31.6–34.7	< 0.0001
Ever married	3.9 (34)	2.8–5.4	12.3 (189)	10.7–14.0	6.9 (247)	6.1–7.7	0.596
Educational Level							
None	2.1 (18)	1.3–3.3	3.7 (57)	2.9–4.8	3.1 (112)	2.9–3.7	
Primary	19.2 (168)	16.7–22.0	20.0 (309)	18.1–22.1	13.9 (500)	12.8–15.0	
Secondary	63.3 (553)	60.0–66.4	50.3 (777)	47.8–52.8	55.4 (2000)	53.8–57.0	
Tertiary	15.5 (135)	13.2–18.0	26.0 (402)	23.9–28.3	27.7 (999)	26.2–29.2	< 0.0001
§Sexual partners in past 6 months							
Median No. of insertive anal sex partners (IQR)	2 (1–4)		2 (0–4)		2 (0–3)		0.0001
Median No. of receptive anal sex partners (IQR)	2 (1–4)		1 (0–3)		1 (0–3)		0.0001
Median No. of partners who paid for sex (IQR)	0 (1–4)		0 (0–2)		0 (0–1)		0.0001
Median No. of non commercial sex partners (IQR)	3 (1–5)		1 (0–3)		1 (1–1)		0.0001
Had female sex partner	15.2 (134)	13.0–17.8	52.2 (785)	49.7–54.7	72.4 (2537)	70.9–73.8	< 0.0001
Engaged in transactional sex	32.7 (276)	29.6–35.9	38.8 (555)	36.3–41.3	33.2 (1130)	31.6–34.8	0.161
Consistent condom use during sex in the past 6 month							
Consistent condom use when selling sex	35.0 (42)	27.0–44.0	52.1 (96)	44.9–59.3	78.6 (265)	73.9–82.7	< 0.0001
Consistent condom use when buying sex	35.2 (95)	29.7–41.1	34.2 (176)	30.2–38.4	61.3 (669)	58.4–64.2	< 0.0001
Consistent condom use with non-commercial partners	32.2 (236)	28.9–35.6	34.9 (320)	31.8–38.0	50.8 (1406)	48.9–52.6	< 0.0001
Sexual position in past 6 months							
Engaged in receptive sex only	20.5 (179)	18.0–23.3	27.7 (423)	25.5–30.0	23.8 (852)	32.1–35.2	
Engaged in insertive sex only	24.4 (213)	21.7–27.4	38.4 (586)	36.0–40.9	33.7 (1206)	32.1–35.2	
Engaged in both insertive and receptive sex	55.1 (480)	51.7–58.3	33.9 (517)	31.5–36.3	42.6 (1525)	41.0–44.2	0.002
HIV risk and exposure to interventions							
Ever tested for HIV	34.0 (299)	31.0–37.2	58.6 (777)	55.9–61.2	64.6 (2312)	63.0–66.2	< 0.0001
Tested for HIV within 1 years of survey	72.9 (218)	67.6–77.7	77.1 (533)	73.9–80.1	78.9 (2327)	77.2–80.6	0.019
Feels at risk to HIV	32.3 (278)	29.2–35.4	27.8 (322)	25.3–30.4	27.6 (990)	26.2–29.1	0.055
Experienced an STI in the past 6 months	6.8 (60)	5.3–8.7	15.0 (232)	13.3–16.9	18.6 (670)	17.3–19.9	0.039

§Sexual position as defined by the sexual position (insertive, receptive or both) practiced by the respondent. *p* values derived from Cochran-Armitage Chi-square trend test

### Sexual risk Behaviours

The median number of insertive anal partners was consistent between 2007 and 2010 with about 50% of the respondents reporting insertive anal sex (IAS) with at least two partners interquartile range ([IQR] 1–4) in the last 6 months. For receptive anal sex (RAS), a median of two sexual partners was reported in 2007 while it was one partner in both 2010 and 2014. Overall, about a third had engaged in transactional sex in 2007 (95% confidence interval (CI):30–36%) and 2014 (32–35%), while it was about two-fifths in 2010 (36–41%). Consistent condom use, when sex was sold increased steadily from 35% (27–44%) to 79% (74–83%) between 2007 and 2014. Similarly, consistent condom use in the past 6 months prior to the survey during paid sexual encounters increased from 35% (30–41%) in 2007 to 61% (58–64%) in 2014. For non-commercial partners, consistent condom use increased from 32% (29–36%) in 2007 to 51% (49–53%) in 2014. For self-reported experiences of sexually transmitted infection (STI), there was an increase from 7% (5–9%) in 2007 to 15% (13–17%) in 2010 and 19% (17–20%) in 2014.

### HIV risk perception and exposure to HIV interventions

Only about a third of respondents (32%; 95% CI:29–35%) felt they were at risk to HIV in 2007. This further decreased to 28% (25–30%) in 2010 and remained at 28% (26–29%) in 2014 with borderline significance (*p* = 0∙055). Self-reported previous HIV test increased steadily from 34% (31–37%) in 2007 to 59% (56–61%) in 2010 and 65% (63–66%) in 2014. An assessment of the recency of the HIV test showed that over 70% reported that they received their HIV test within 1 year of the study, with the highest recent tests reported in 2014 (79%; 77–81%).

### Change in HIV prevalence

As shown in Table 1, HIV prevalence increased steadily between 2007 and 2014. From 14% (11–16%) in 2007, it increased to 17% (15–19%) in 2010 and 23% (22–34%) in 2014. Table 2 shows HIV prevalence disaggregated by risk behaviors. When analysis was restricted to only states that participated in the three rounds of the IBBSS (Cross River, Kano and Lagos) HIV prevalence was 13% (11–16%) in 2010 and 25% (22–27%) in 2014.

**Table 2:** HIV prevalence disaggregated by sociodemographics and sexual risk behaviours

Characteristics	2007% (n)	95%CI	2010% (n)	95%CI	2014% (n)	95% CI	*p* value
Age (years)							
16–19	13.3 (13)	7.8–21.6	12.0 (24)	8.2–17.3	12.4 (82)	10.1–15.1	0.909
20–24	8.9 (44)	6.7–11.7	16.2 (87)	13.3–19.6	21.1 (303)	19.1–23.3	< 0.0001
> =25	24.1 (52)	18.8–30.2	20.0 (111)	16.9–23.6	32.3 (326)	29.5–35.3	< 0.0001
Engaged in receptive sex only in past 6 months	19.0 (31)	13.7–25.8	23.1 (76)	18.9–28.0	24.4 (187)	21.5–27.6	0.177
Engaged in insertive sex only in past 6 months	11.3 (23)	7.6–16.5	14.1 (69)	11.2–17.5	18.6 (193)	16.3–21.1	0.002
Engaged in both insertive and receptive sex in past 6 months	12.4 (54)	9.6–15.8	16.0 (73)	12.9–19.7	25.1 (321)	22.8–27.6	< 0.0001
Engaged in transactional sex in past 6 months	14.6 (37)	10.7–19.5	18.7 (89)	15.4–22.4	18.7 (191)	16.5–21.3	0.218
Feels at risk to HIV	18.9 (49)	14.6–24.2	24.1 (60)	19.2–29.8	27.3 (238)	24.4–30.4	0.006
Experienced STI symptoms in past 6 months	10.5 (6)	4.8–21.7	21.3 (43)	16.2–27.5	25.0 (151)	21.7–28.7	0.019
Had female sex partner in past 6 months	10.2 (13)	6.0–16.9	16.6 (108)	13.9–19.6	23.3 (494)	21.5–25.1	< 0.0001
States							
Cross River	2.8 (8)	1.4–5.4	5.7 (14)	3.6–9.1	11.3 (52)	8.7–14.5	< 0.0001
Enugu	NA		NA		16.8 (69)	13.5–20.8	
FCT	NA		44.4 (55)	35.8–53.2	30.1 (169)	26.4–34.0	0.002
Kaduna	NA		23.1 (48)	17.8–29.3	15.5 (76)	12.5–19.0	0.016
Kano	11.7 (27)	8.2–16.6	11.4 (31)	8.1–15.7	14.9 (13)	8.8–24.1	0.514
Lagos	25.4 (74)	20.7–30.8	27.1 (52)	21.2–33.8	41.4 (172)	36.7–46.2	< 0.0001
Oyo	NA		9.6 (19)	6.2–14.6	14.2 (62)	11.2–17.8	0.105
Rivers	NA		NA		40.7 (98)	34.6–47.0	

*P* value derived from Chocran_Armitage Chi-square trend tests. *CI* Confidence interval

Among those who reported RAS only, HIV prevalence was 19% (14–26%) in 2007, 23% (19–28%) in 2010 and 24% (22–28%) in 2014. By age group, HIV prevalence was stable among those aged 16–19 years (*p* = 0∙953) while it increased among those aged 20–24 years, from 9% (7–12%) in 2007 to 21% (19–23%) in 2014.

For the six states with data from at least two rounds of IBBSS, there was increase in HIV prevalence in four of the states between the two rounds, while two states recorded declines. For Cross River state, HIV prevalence increased from 3% (1–5%) in 2007 to 6% (4–9%) in 2010 and 11% (9–15%) in 2014 while in FCT (44% [36–53%] vs. 30%; [26–34%] and Kaduna state (23% [18–29%] vs. 16% [13–19%]) there was about 30% decrease in HIV prevalence between 2010 and 2014.

### Factors associated with HIV prevalence among MSM

Table 3 outlines factors associated with HIV among MSM in Nigeria. When compared to MSM aged 16–19 years, those aged 20–24 years and ≥ 25 years were more likely to be HIV positive (adjusted odds ratio [AOR] 1∙40; 95% CI: 1∙09–1.80) and (AOR 2∙41; 95% CI:1∙84–3∙16) respectively. Compared to those who engaged in IAS only, those who engaged in RAS only (AOR 1∙68; 95% CI:1∙11–2∙54) or both IAS and RAS (AOR 1.71; 95% CI:1.40–2.10) were more likely to be HIV positive. With Cross River state as the reference, MSM in Enugu state (AOR 1.89; 95% CI: 1∙26–2.80), FCT (AOR:4.23; 95% CI:3.04–5.87), Kaduna state (AOR: 2.27; 95% CI: 1∙59–3.23), Kano state (AOR: 1.97; 95% CI: 1∙29–3.00), Lagos state (AOR:6.66; 95% CI: 4.93–8.99) and Rivers state (AOR: 7.37; 95% CI:4∙96–10.94) were more likely to be HIV positive. Education and transactional sex were not associated with HIV among MSM in Nigeria.

**Table 3:** Multivariate analysis showing factors associated with HIV among MSM in Nigeria

Factors	Crude OR (95% CI)	*p* value	Adjusted OR (95% CI)	*p* value
Age (years)				
16–19	1		1	
20–24	1∙51 (1∙21–1∙88)	< 0∙0001	1∙40 (1∙09–1∙80)	0∙008
> =25	2∙68 (2∙15–3∙33)	< 0∙0001	2∙41 (1∙84–3∙16)	< 0∙0001
Ever married				
No	1			
Yes	0∙97 (0∙75–1∙26)	0∙817		
Educational level				
None	1		1	
Primary	0∙6 (0∙40–0∙91)	0∙015	0∙87 (0∙53–1∙42)	0∙571
Secondary	0∙79 (0∙54–1∙15)	0∙218	1∙02 (0∙65–1∙61)	0∙917
Tertiary	1∙25 (0∙85–1∙84)	0∙255	1∙16 (0∙73–1∙84)	0∙534
Sexual position in past 6 months				
Insertive sex only	1		1	
Receptive only	1∙55 (1∙29–1∙86)	< 0∙0001	1∙92 (1∙54–2∙40)	< 0∙0001
Both	1∙32 (1∙12–1∙56)	0∙001	1∙71 (1∙40–2∙10)	< 0∙001
Engaged in transactional sex in past 6 months				
No	1		1	
Yes	0∙86 (0∙74–0∙99)	0∙046	0∙91 (0∙77–1∙09)	0∙308
Consistent condom when paid for sex in past 6 months				
Always	1			
Sometimes	0∙76 (0∙42–1∙36)	0∙353		
Feels at risk to HIV				
No	1		1	
Yes	1∙06 (1∙01–1∙12)	0∙012	1∙36 (1∙14–1∙61)	< 0∙0001
Don’t know			0∙96 (0∙59–1∙54)	0∙852
Experienced STI symptoms in past 6 months				
No	1		1	
Yes	1∙26 (1∙05–1∙50)	0∙011	1∙26 (1∙02–1∙55)	0∙034
Had female sexual partner in past 6 months				
No	1		1	
Yes	1∙23 (1∙07–1∙42)	0∙004	0∙93 (0∙77–1∙12)	0∙439
States				
Cross River	1		1	
Enugu	2∙54 (1∙80–3∙60)	< 0∙0001	1∙89 (1∙26–2∙80)	0∙002
FCT	6∙10 (4∙60–8∙08)	< 0∙0001	4∙23 (3∙04–5∙87)	< 0∙0001
Kaduna	2∙71 (2∙00–3∙67)	< 0∙0001	2∙27 (1∙59–3∙23)	< 0∙0001
Kano	1∙72 (1∙22–2∙42)	< 0∙002	1∙97 (1∙29–3∙00)	0∙002
Lagos	6∙23 (4∙76–8∙17)	< 0∙0001	6∙66 (4∙93–8∙99)	< 0∙0001
Oyo	1∙84 (1∙32–2∙56)	< 0∙0001	1∙65 (1∙13–2∙39)	0∙009
Rivers	8∙62 (6∙09–12∙18)	< 0∙0001	7∙37 (4∙96–10∙94)	< 0∙0001
Year				
2007	1		1	
2010	1∙34 (1∙04–1∙71)	0∙022	1∙92 (1∙40–2∙62)	< 0∙0001
2014	1∙91 (1∙54–2∙38)	< 0∙0001	2∙04 (1∙51–2∙75)	< 0∙0001

*OR* Odds ratio, *CI* Confidence interval

## Discussion

This is the first study to conduct a trend analysis of HIV prevalence and its correlates among MSM in Nigeria and we identified several important findings. First, HIV prevalence has steadily increased over time with a 10-percentage point increase every year over 7 years. Second, the burden of HIV is higher among older MSM than younger ones. Third, prevalence of STI has also increased over the years and has more than doubled from 7% in 2010 to 17% in 2014. Fourth, although consistent condom use has increased with transactional sex, the increase is less with non-transactional sex. Fifth, less than 70% of MSM have ever been tested for HIV highlighting major gaps in HIV prevention intervention for MSM. Sixth, only about a third of MSM felt they were at risk for HIV and lastly, compared to Cross River states, MSM who reside in other states except Kano state were more likely to be HIV positive. These findings directly mirror the state of HIV programming for MSM in Nigeria and strategies, policies and programs must be designed to address these gaps.

Between the first and second rounds of IBBSS in 2007 through 2010, female sex workers (FSW) had shown the highest prevalence of HIV among key populations in Nigeria. However, in the third round of IBBSS in 2014, HIV prevalence among MSM (23%) exceeded that of FSW (19%) which has been on a decline from 37% to 19% and 30 to 9% among brothel and non-brothel based female sex workers respectively [25]. The relative increase in HIV incidence among MSM in the era of expanded ART and in which there’s been HIV decline among other groups has been termed “resurgent epidemic in MSM” [1, 4, 26, 27] and future studies among this group may benefit from incidence studies to estimate the HIV incidence rate among MSM in Nigeria. Unprotected anal intercourse (UAI) remains the main risk factor for HIV among MSM and studies have demonstrated the high transmission efficiency of HIV through anal sex [1]. Kingsley et al. (1987) reported a 20-fold increased risk of HIV seroconversion over 6 months among MSM who reported UAI when compared to those who did not [4, 28]. Baggaley et al., (2008) in a systematic review and meta-analysis of HIV transmission risks in anal sex, reported a 1.4% transmission probability per-act of unprotective receptive anal intercourse (URAI) and 40∙4% (6∙0–74∙9) per-partner probability, with no difference between MSM and heterosexual anal intercourse [29]. The 1.4% per-act probability for URAI has been estimated to be roughly 18-times greater than that of vaginal intercourse [1, 30]. An updated review in 2018, showed a pooled HIV-1 risk of 1.3% for URAI with no difference between the pre-ART and ART era (1.7% vs. 0.8%; *p* = 0.537) [31]. Findings from our study showed that those who engaged in receptive anal sex only, were twice more likely to be HIV positive compared to those who reported only insertive anal sex. Similarly, those who engaged in both insertive and receptive anal sex were twice as likely to be HIV positive when compared to only those who practiced insertive anal sex. This corroborates Baggaley et al’s (2008) findings in their systematic review where per-partner risks for infection were similar for people reporting exclusive unprotected receptive anal intercourse and both unprotected receptive and insertive anal intercourse [1, 29]. These factors have been suggested as key drivers of the rapid and efficient spread of HIV through networks of MSM [2].

Consistent condom use provides about 70–80% efficacy in preventing HIV transmission [2, 32–34]. In this study, consistent condom use increased from 2007 to 2014, when sex was sold or bought and with non-transactional partners. However, while consistent condom use in past 6 months more than doubled when sex was sold, only 50% of those who reported non-transactional sex used condoms consistently. This suggests that within MSM sexual networks, there’s an increased probability of HIV transmission which may negate the increased use of condom during transactional sex. Sero-adaptation, including serosorting and strategic or sero-positioning, which rely on knowing one’s HIV status as well as that of their sex partners, have been used by MSM as prevention approaches [1, 35]. Serosorting involves the selection of HIV-concordant sex partners, while sero-positioning involves choosing sex acts based on serostatus [1]. A study in Seattle, U.S.A, showed that among recently infected MSM, 69% reported UAI with HIV-positive or unknown status partners compared with 32% in HIV uninfected controls [4, 36]. The Swiss HIV cohort study reported that the strongest predictor of UAI was knowing the HIV status of sexual partners with consistent condom use being 89% between stable discordant couples and 48% between HIV-infected partners [4, 37]. The role of seroadapation in Nigeria is unknown and more so with less than 70% of MSM ever being tested for HIV, it’s unlikely that this practice is widespread as the knowledge of HIV status of partners remains limited within networks.

The low consistent condom use in non-transactional sex may explain the significant increase in self-reported STI between 2007 and 2014. Furthermore, among those who reported STIs, HIV prevalence increased between 2007 and 2014. Higher prevalence of STIs and undiagnosed HIV infections are markers of suboptimal access to clinically competent and appropriate health care services which are in turn reported to reduce HIV-related health-seeking behaviour in African MSM [1, 38]. The suboptimal access to healthcare and discrimination by healthcare workers are further worsened by the poor funding of MSM targeted prevention and treatment services in Nigeria. Between 2007 and 2012, less than 5% of HIV funding was dedicated to most-at-risk population and less than 5% dedicated to enabling environment for HIV programs [39–41].

The increase in consistent condom use observed during transactional sex may explain the low perceived risk of HIV among MSM. Less than a third of MSM felt at risk of HIV and given the increased prevalence of HIV and STIs among MSM in Nigeria, efforts must be made to heighten HIV risk perception. The psychometric paradigm theory and a number of other social and health psychology theories [42–49] have identified risk perception as having a central role in determining behavior. A meta-analysis of risk appraisal reported that interventions that successfully heightened the risk appraisal within an individual, resulted to changes in subsequent intentions and behaviour [50]. Similarly, de Hoog et al. (2007) reported that when the severity of a threat was heightened, irrespective of the channel of communication, there was an associated positive and significant effect on intention and behaviour change [51]. Behaviour change interventions for HIV programs should be designed to heighten the threat of HIV.

Older MSM were more likely to be HIV positive than younger MSM. HIV prevalence among MSM aged 16–19 years remained unchanged between 2007 and 2014 and was lower than that reported for those aged 20–24 years and those 25 years and above. Merrigan et al. (2010) reported similar results among MSM in three states in Nigeria [52]. Another recent study in Nigeria among MSM who engaged in transactional sex, showed that MSM aged 25 years and above were four times more likely to be HIV positive than those aged 15–19 years [53]. However, our findings are contrary to those reported by Beyrer et al. (2012) and (2016) which indicated that younger MSM had higher burden of HIV [1, 54]. A plausible explanation for our finding is that the older MSM have had prolonged exposure to HIV through higher number of sexual partners, engaging in transactional sex and higher exposure to unprotected anal sex. Furthermore, MSM sampled in 2010 and 2014 were more likely to be HIV positive compared to those in 2007 and this further supports our argument that the prolonged exposure to higher risk behaviours may be the reason behind higher HIV prevalence among older MSM. Younger MSM aged 16–19 years are likely to still have parental support and thus, there’s less socioeconomic pressure to engage in high risk sexual practices such as transactional sex. In addition, their sexual networks revolve around their peers rather than intergenerational sexual partners and this limits their exposure to older HIV infected MSM.

There was a significant increase in the proportion of MSM who reported having sex with female partners between 2007 and 2014. This constitutes a potential bridge between MSM and the general population and thus merits discussion as the gains in reduction of HIV prevalence among the general population may be eroded by bisexual intercourse among MSM. The increase in bisexuality may reflect the increasing hostility, stigma and criminalization of MSM in Nigeria. Schwartz et al. (2015) assessed the immediate effects of the same-sex marriage prohibition act in Nigeria and reported a statistically significant increase in proportion of MSM who had female partners after the law was passed when compared to the pre-law period [13]. This coping mechanism to the high stigma and criminalization of MSM may also negatively impact their utilization of key population friendly clinics as they continue to hide their identity even to health care workers.

This study has some limitations. The absence of a prospective study group and the use of cross-sectional surveys from unmatched cohort limits the strength of our study and thus requires caution in the interpretation of the data. There may be potential dependence between data from different rounds of IBBSS which may overestimate HIV prevalence if a significant number of positives from previous rounds were recruited into subsequent rounds or an underestimation of HIV prevalence is a significant number of HIV negative MSM were targeted and recruited in subsequent rounds. Future studies should include a variable to help identify those in previous rounds and their HIV status at that round to allow a more robust estimation of HIV among MSM. Data on HIV prevention programs and treatment coverage in the study states was not available and thus could not be accounted for in our study to independently measure the impacts of these programs in the study outcome. In addition, data on treatment coverage could help explain the observed increase if treatment coverage was assessed to be low. Another limitation is that of social desirability bias on sexual risk behaviours as information were self-reported, however the higher increase in consistent condom use during transactional sex compared to non-transactional is comparable to that observed among female sex workers [54] and suggests that risks behaviors captured in these studies may have been under-reported given the increase of STIs and HIV observed. Furthermore, studies on biological validation of unprotected sex among female sex workers have shown significant over-reporting of protected sex [55] and future studies should consider biological validation of protected sex among MSM to better characterize risk behaviours Drug use especially use of methamphetamine [1] has been associated with HIV among MSM, however, there was no data on drug use among MSM in all three rounds of the survey. Further research is required to determine the association of drug use and HIV among MSM in Nigeria. Lastly, not all clients opted for an HIV test and the proportion of refusal ranged from 10 to 16%. While the status of those who rejected an HIV test cannot be assumed, participants who refused to opt for an HIV test may have done so because of previous knowledge of HIV infection and thus prevalence of HIV may have been underestimated in the current study and subsequently biases the observed trend in HIV prevalence.

## Conclusions

In conclusion, this the first study to evaluate the trend of HIV prevalence among MSM in Nigeria and we report a number of key observations. As in other climes, HIV prevalence among MSM in Nigeria is on an alarming progression with a relative increase of 10% point per year over 7 years. No state is spared, and prevention packages must be holistic and involve the use of strategies with the strongest evidence of highest efficacy in preventing HIV transmission; early treatment of partners, [2, 56] condoms [32, 57] and oral preexposure prophylaxis [58]. Lastly, the HIV epidemic among MSM in Nigeria is severe and clearly, is one of the defining challenges ahead, and maybe the most critical gap in the national HIV prevention program to control the HIV epidemic in Nigeria.

## Data Availability

Data are available under reasonable request from the corresponding author.

## Abbreviations

AOR: Adjusted Odds Ratio
ART: Antiretroviral Treatment
CI: Confidence Interval
FCT: Federal Capital Territory
FSW: Female Sex Workers
HIV: Human Immunodeficiency Virus
IAS: Insertive Anal Sex
IBBSS: Integrated Biological and Behavioral Surveillance Survey
MSM: Men who have sex with men
RAS: Receptive Anal Sex
RDS: Respondent Driven Sampling
STI: Sexually Transmitted Infections
UAI: Unprotected Anal Intercourse
URAI: Unprotected Receptive Anal Intercourse
USD: United States Dollars
